# Expression of mammalian mitochondrial F_1_‐ATPase in *Escherichia coli* depends on two chaperone factors, AF1 and AF2

**DOI:** 10.1002/2211-5463.12143

**Published:** 2016-10-25

**Authors:** Toshiharu Suzuki, Naoya Iida, Junko Suzuki, Yasunori Watanabe, Toshiya Endo, Toru Hisabori, Masasuke Yoshida

**Affiliations:** ^1^ Faculty of Science and EngineeringWaseda UniversityTokyoJapan; ^2^ Department of Molecular BioscienceKyoto‐Sangyo UniversityKyotoJapan; ^3^ Chemical Resources LaboratoryTokyo Institute of TechnologyYokohamaJapan; ^4^ Present address: Department of Applied ChemistrySchool of EngineeringThe University of TokyoHongo, Bunkyo‐kuTokyo113‐8656Japan

**Keywords:** F_1_‐ATPase, F_o_F_1_‐ATP synthase, molecular chaperone

## Abstract

F_1_‐ATPase (F_1_) is a multisubunit water‐soluble domain of F_o_F_1‐_ ATP synthase and is a rotary enzyme by itself. Earlier genetic studies using yeast suggested that two factors, Atp11p and Atp12p, contribute to F_1_ assembly. Here, we show that their mammalian counterparts, AF1 and AF2, are essential and sufficient for efficient production of recombinant bovine mitochondrial F_1_ in *Escherichia coli* cells. Intactness of the function and conformation of the *E. coli*‐expressed bovine F_1_ was verified by rotation analysis and crystallization. This expression system opens a way for the previously unattempted mutation study of mammalian mitochondrial F_1_.

**Abbreviations**
AMPPNP: adenylyl‐imidodiphosphate
Au‐bead: colloidal gold particles
CBB: Coomassie Brilliant Blue
F_1_: F_1_‐ATPase
F_o_F_1_: F_o_F_1_‐ATP synthase
fps: frames per second
IF1: inhibitory factor‐1
Pi: inorganic phosphate
rps: revolutions per second

F_o_F_1_‐ATP synthase (F_o_F_1_) is ubiquitously found in membranes of bacteria, chloroplasts, and mitochondria, and synthesizes ATP from ADP and inorganic phosphate (Pi) driven by downhill proton flow across the membranes 1, 2, 3. F_1_‐ATPase (F_1_) is a water‐soluble catalytic domain of F_o_F_1_, which has a subunit composition of α_3_β_3_γδε. F_1_ is a rotary motor, where net hydrolysis of one ATP molecule drives a 120° rotation of a central rotor shaft composed of γδε‐subunits relative to a surrounding stator ring of α_3_β_3_‐subunits (eukaryotic subunit composition) 4, 5. Extensive studies on rotation of bacterial F_1_ revealed six‐step rotation in one revolution, that is, repetition of an 80° rotation by ATP binding to one of the three catalytic β‐subunits and a 40° rotation by release of Pi from another β‐subunit 4, 6, 7, 8, 9. Understanding the rotation mechanism of F_1_ requires knowledge on how chemical events occurring to F_1_ induce its structural changes and trigger rotation. In this respect, much structural information has been accumulated for bovine F_1_, rather than bacterial F_1_, by X‐ray crystallography 10, 11. However, rotation of mitochondrial F_1_ was not demonstrated until recently because of the absence of *in vitro* expression system of mitochondrial F_1_ genes that enables genetic modification necessary for single‐molecule observation, such as introduction of the His‐tag.

We recently succeeded in expressing human mitochondrial F_1_ in *Escherichia coli* cells and reported its nine‐step rotation in one revolution 5. Following up this work, here, we report the expression of bovine mitochondrial F_1_ in *E. coli*. Early genetic works using *Saccharomyces cerevisiae* identified two mitochondrial proteins of Atp11p and Atp12p as molecular chaperones necessary for assembly of F_1_ 12. Analyses using a yeast two‐hybrid system and immunoprecipitation further showed direct interaction of Atp11p 13 with a β‐subunit and of Atp12p with an α‐subunit 14. Mammalian homologs of these chaperones are ATPAF1 and ATPAF2 (AF1 and AF2, hereafter), and their coding genes, *ATP11* and *ATP12*, can respectively complement genetic deficiencies of *ATP11* 15 and *ATP12* 16 of yeast. AF1 and AF2 have antiaggregation activity toward reduced insulin 17, 18 and citrate synthase *in vitro*, respectively 19. However, whether AF1 and AF2 are essential for the production of mammalian F_1_ was not tested directly. We thus expressed the five subunits of bovine mitochondrial F_1_ in *E. coli* cells with or without coexpression of AF1 and AF2. The results clearly show that AF1 and AF2 are essential and sufficient for the production of bovine F_1_ in *E. coli*. ATP‐driven rotation and crystallization confirmed intactness of *E. coli*‐expressed bovine F_1_.

## Experimental procedures

### Expression of bovine F_1_ in *E. coli*

The expression plasmid for bovine F_1_ was constructed in the same manner as performed previously for human F_1_ 5; five genes coding subunits of bovine F_1_ (α, β, γ, δ, and ε) 20 and two genes, *ATP11* and *ATP12*, were amplified by PCR from the cDNA library prepared from the total RNA of bovine heart muscle. The genes were tandemly introduced in the order of α‐γ‐β‐δ‐ε‐*ATP11*‐*ATP12* into the expression vector pTR19 21, which are transcribed from the *trc* promoter. A histidine tag composed of 10 histidine residues was genetically introduced into the N terminus of the β‐subunit of F_1_ as performed previously 4. The resulting plasmid, pBF1, was introduced into F_o_F_1_‐deficient *E. coli* strain, DK8 21. The recombinant *E. coli* strain was cultivated in 2 × YT medium containing 100 μg·mL^−1^ ampicillin for 40 h at 29 °C. The culture flasks were shaken for aeration because respiration of cells is necessary for efficient expression even though growth is dependent on glycolysis. As observed in the case of expression of F_1_ from thermophilic *Bacillus* PS3 in *E. coli* 22, the growth rate of the *E. coli* was not significantly affected by the expression of bovine F_1_. It is assumed that submillimolar concentration of ADP in cytoplasm is enough to keep F_1_ in the inactive state of so‐called MgADP‐inhibition, a general feature of F_1_ from any sources 23. The cells were disrupted and the water‐soluble fraction was subjected to Ni‐affinity column chromatography and gel‐filtration column chromatography. Purification procedures for bovine F_1_ are the same as those for human F_1_, except the buffers for cell lysis (20 mm potassium phosphate (pH 7.5), 100 mm KCl and 0.1 mm ATP) and for gel‐filtration (40 mm Tris/HCl (pH 8.0), 200 mm NaCl, 1 mm EDTA and 0.1 mm ATP). After gel‐filtration with Superdex200 10/300GL column (GE Healthcare, Uppsala, Sweden), fractions of a peak having the ATPase activity were collected, concentrated with a centrifugal concentrator (50 kDa, Centricon50; Millipore Corp., Billerica, MA, USA), and used for further analyses. Yield of the purified recombinant bovine F_1_ was about 2–3 mg per 6‐L‐culture. Authentic bovine F_1_ was prepared from bovine heart as reported 24 with a modification; gel‐filtration was performed with a Superdex200 column in 20 mm Tris/HCl (pH8.0), 200 mm NaCl, 0.1 mm ATP, and 0.5 mm EDTA. To avoid cold dissociation of bovine F_1_, all procedures were carried out at a temperature higher than 20 °C. Mutated IF1 (IF1‐GFP) used in this study, I60GFPHis, was prepared as reported previously 25.

### Rotation of *E. coli*‐expressed bovine F_1_

Rotation of a single molecule of bovine F_1_ was observed by the procedures described in ref. 5. Two cysteine residues were introduced into a globular domain of γ‐subunit (γAla99Cys and γSer191Cys). Images of a rotating submicron polystyrene bead attached to the γ‐subunit of immobilized bovine F_1_ were captured with a CCD camera (ICL‐B0620M; Implex, Minneapolis, MN, USA) at 500 frames per sec (fps) under illumination of a mercury lamp. Rotation of the Au‐bead (40 nm diameter) was observed at 25 000 fps with a laser‐illuminated center‐shielded dark‐field microscopic system equipped with a high‐speed camera (MEMRECAM GX‐8S; NAC Image Technology Inc., Tokyo, Japan) 5.

### Crystallization of *E. coli*‐expressed bovine F_1_

Concentrated recombinant bovine F_1_ was supplemented with 0.5 mm AMPPNP and 20 mm MgCl_2_ (the final bovine F_1_ concentration was 10 mg·mL^−1^) and used for crystallization. Reservoir solution (70 μL) containing 100 mm Tris/HCl (pH 8.5), 200 mm LiSO_4_, and 21–23% PEG3350 (Hampton Research, Aliso Viejo, CA, USA) was put into a sitting‐drop dish, and the bovine F_1_ solution and the reservoir solution (each 0.25 μL) were mixed to make one sitting drop. Crystals with the size of 0.05–0.3 mm were grown in approximately 3 weeks at 20 °C. For analysis of the crystals with polyacrylamide gel electrophoresis in the presence of sodium dodecylsulfate (SDS/PAGE), 10–20 crystals were collected from crystallization drops using a cryoloop, washed four times with 100 μL of wash solution (the reservoir solution supplemented with 0.5 mm AMPPNP, 20 mm MgCl_2_, and 25 mm NaCl), and dissolved in the SDS/PAGE sample buffer. After electrophoresis, the gel was stained with silver.

### Other methods

The ATPase activity was measured in 50 mm HEPES/KOH buffer (pH 7.5) containing 100 mm KCl, 1 mm MgCl_2_, 1 mm ATP, and the ATP‐regenerating system 21 supplemented with 0.2 mm NADH and 0.2 mg·mL^−1^ lactate dehydrogenase 26. The reaction was initiated by adding F_1_, and the change in absorbance at 340 nm was recorded. The ATPase activity was calculated from the slope of absorbance decrease during 400–500 s. For the assay of IF1 inhibition, IF1‐GFP was added to the reaction mixture prior to the measurement at the indicated concentration. Previous studies of authentic bovine F_1_ 25 showed that IC_50_ of IF1‐GFP is 65 nm, while that of wild‐type IF1 is approximately 10 nm 5. Protein concentrations were determined by protein assay kit (Pierce Biotechnology Inc., Rockford, IL, USA), with bovine serum albumin as a standard. All SDS/PAGE and native‐PAGE in this study were performed with a gradient polyacrylamide gel (10–20%) and nongradient gel (12%). The proteins were visualized by Coomassie Brilliant Blue (CBB) or by immunoblotting with anti‐β and anti‐δ antibodies. All data used for this study were measured at least in triplicate.

## Results and Discussion

### *Escherichia coli* expression of bovine F_1_ depends on AF1 and AF2

**Figure 1:**
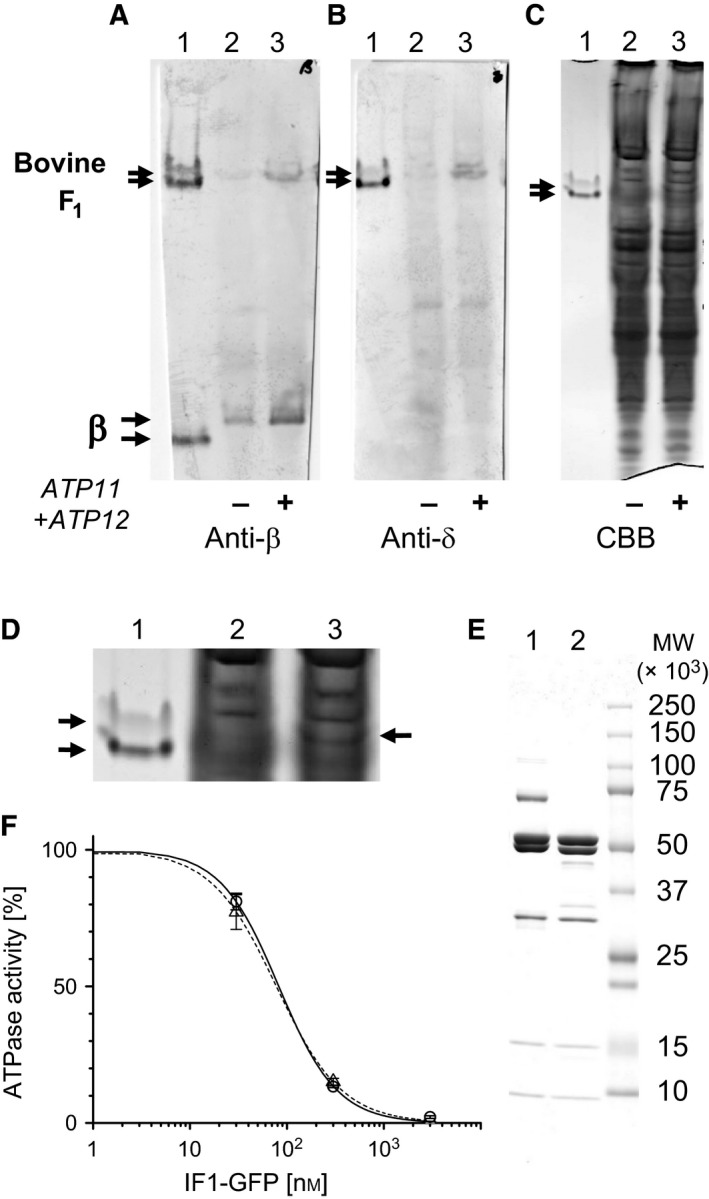
Expression of bovine F_1_ in *Escherichia coli*. (A–D) Native‐PAGE analysis of water‐soluble fraction of *E. coli* cells expressing bovine F_1_ without (plasmid pBF1(‐AFs), lane 2) or with (pBF1(+AFs), lane 3) simultaneous expression of the two genes, *ATP11* and *ATP12*. Lane 1 represents authentic bovine F_1_ purified from bovine heart muscle. The gels were analyzed by immunoblotting using anti‐β‐subunit (A) or anti‐δ‐subunit antibodies (B), or by protein staining with CBB (C, D). (D) The region containing a band of bovine F_1_ in C is enlarged. Arrows indicate the band of bovine F_1_. (E) SDS/PAGE analysis of authentic bovine F_1_ (lane 1) and purified *E. coli*‐expressed bovine F_1_ (lane 2). (F) Inhibition of ATPase activity of bovine F_1_ by IF1. GFP‐fused IF1 was used. The ATPase activity in the absence of IF1‐GFP is set to 100%. Solid line, *E. coli*‐expressed bovine F_1_; dotted line, authentic bovine F_1_.

The five genes for bovine F_1_ were introduced into the *E. coli* expression vector in the same order as in the *E. coli* F_o_F_1_ operon, α‐γ‐β‐δ‐ε, to generate a plasmid pBF1(‐AFs). A set of genes, *ATP11* and *ATP12*, were further introduced at the end of the operon as α‐γ‐β‐δ‐ε‐*ATP11‐ATP12* to generate a plasmid pBF1(+AFs). These plasmids were individually introduced into the *E. coli* strain that lacks the whole F_o_F_1_ operon in the chromosome, and resultant recombinant strains were cultured. The water‐soluble fraction of harvested cells was analyzed with polyacrylamide gel electrophoresis in the absence of SDS (native‐PAGE) using authentic bovine F_1_ purified from bovine heart as a control (Fig. 1A–D). Native‐PAGE followed by immunoblotting with anti‐β antibodies showed that pBF1(+AFs)‐harboring cells produced a significant amount of bovine F_1_ while pBF1(‐AFs)‐harboring cells produced very little, if any, amount of bovine F_1_ (Fig. 1A). The band arising from the monomeric β‐subunit was seen in all samples. The immunoblotting with anti‐δ antibodies confirms pBF1(+AFs)‐dependent production of bovine F_1_ (Fig. 1B). F_1_ isolated from bovine heart appeared as two split bands in native‐PAGE for an unknown reason and bovine F_1_ produced in *E. coli* also gives two bands. The monomeric β‐subunit of bovine F_1_ produced in *E. coli* migrates in the gel more slowly than that of authentic bovine F_1_ due to the attached histidine tag (Fig. 1A). Production of bovine F_1_ in pBF1(+AFs)‐harboring cells was confirmed by protein staining as a faint, but distinct band (Fig. 1C, D). These results show that expression of the *ATP11* and *ATP12* is essential for efficient production of bovine F_1_. We purified bovine F_1_ from pBF1(+AFs)‐harbored *E. coli* cells and confirmed that it has the same subunit composition as authentic bovine F_1_ by SDS/PAGE analysis (Fig. 1E). The ATPase activity of mitochondrial F_1_ is known to be inhibited by a specific inhibitor protein of mitochondria, IF1 25. Sensitivity of *E. coli*‐expressed bovine F_1_ to IF1 was tested by using bovine IF1 fused to GFP. As shown in Fig. 1F, the ATPase activity of *E. coli*‐expressed bovine F_1_ was inhibited by IF1‐GFP in the same manner as observed for authentic bovine F_1_ (Fig. 1F).

### Rotation of *E. coli*‐expressed bovine F_1_

**Figure 2:**
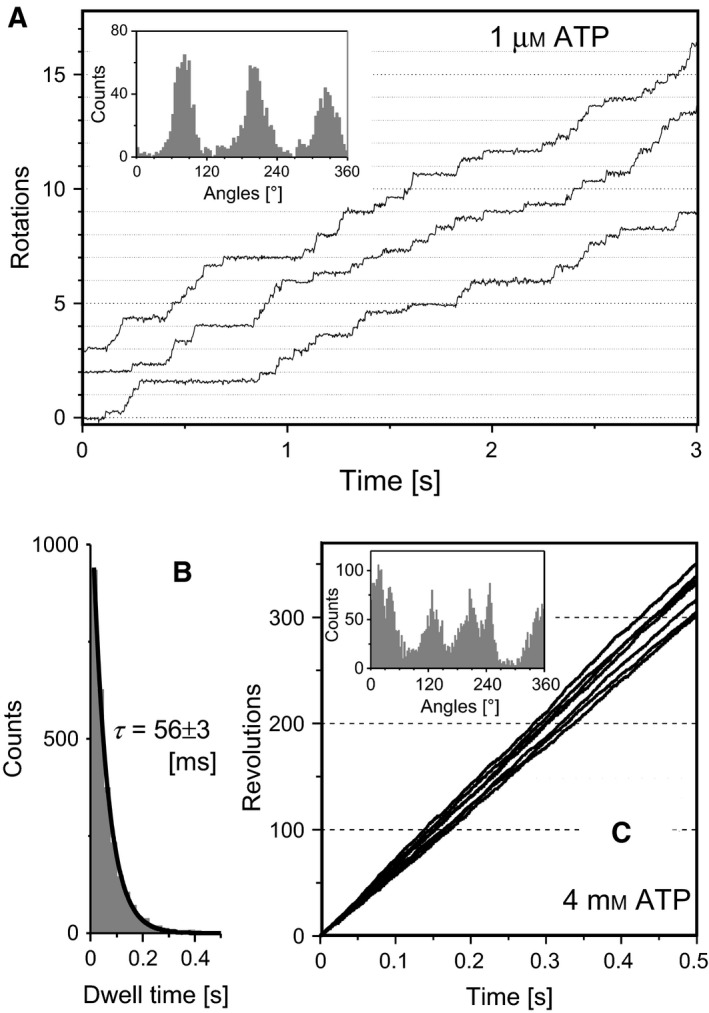
Single‐molecule analysis of rotation of *Escherichia coli*‐expressed bovine F_1_. (A, B) Rotation of bovine F_1_ at 1 μm ATP observed with a camera 500 fps. (A) Time‐courses of the rotation. The inset is an angle histogram of the rotation. (B) A histogram of duration of dwells (*N* = 3672 dwells, three molecules) observed in the rotation. The bin width was 0.25 s. The histogram was best simulated with a single‐exponential decay function with a lifetime of 56 ± 3 ms. (C) Time‐courses of rotation at a saturating ATP concentration, 4 mm. Rotation was analyzed at 25k fps using Au particles (diameter was 40 nm) as a rotation probe. The inset is an angle histogram of the rotation. The averaged rotation speed over 0.5 s was 655 ± 38 rps (*N* = 6 molecules).

To verify the function of *E. coli*‐expressed bovine F_1_, ATPase‐driven rotation was observed by microscopic single‐molecule analysis. For this purpose, a submicron polystyrene bead was attached to two introduced cysteine residues of the γ‐subunit as a rotation probe. At a low ATP concentration (1 μm), bovine F_1_ rotates at a speed 2.5 ± 0.3 rps and rotation takes three dwells per revolution, approximately at every 120° rotation (Fig. 2A). The dwells become shorter as the ATP concentration increased, indicating that bovine F_1_ waits for ATP binding during the dwell to drive the next cycle of 120° rotation. The rate constant of ATP binding (*k*_on_) calculated from the lifetime of the dwell (τ=56 ± 3 ms) is 1.8 ± 0.3 × 10^7^ m ^−1^·s^−1^ (Fig. 2B). Rotation at a saturating ATP concentration (4 mm) was observed with a rapid camera (a frame per 40 μs) (Fig. 2C). By using colloidal gold particles (diameter, 40 nm) as a rotation probe, viscous friction of the rotating particle did not slow down rotation under the experimental conditions and the rotation speed, 655 ± 38 rps (*N* = 7 molecules), directly reflects the maximum turnover rate of ATP hydrolysis by a single molecule of bovine F_1_, that is, ~ 2000 per second. The presence of dwells is suggested from the angle histogram of rotation that awaits extensive analysis. As expected from high sequence conservation between bovine F_1_ and human F_1_, these motor characteristics of bovine F_1_ are similar to those of human F_1_ (*k*_on_, 2.7 ± 0.3 × 10^7^ m ^−1^·s^−1^; rotation speed, 705 ± 75 rps) 5.

### Crystallization of *E. coli*‐expressed bovine F_1_

**Figure 3:**
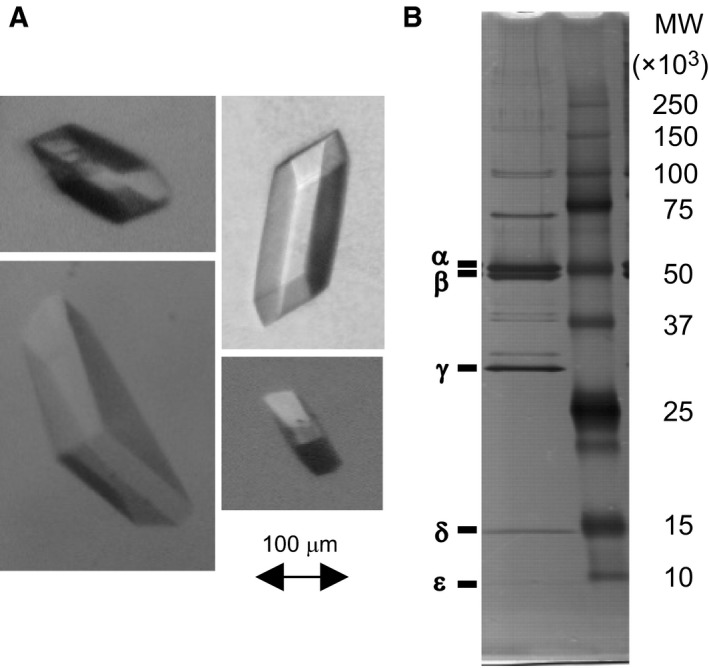
Crystals of *Escherichia coli*‐expressed bovine F_1_. (A) Crystals of bovine F_1_ were routinely obtained by sitting‐drop vapor diffusion method as stated in *Experimental Procedures*. (B) SDS/PAGE analysis of the crystals. The gel was stained with silver.

Bovine F_1_ (without a Cys mutation) purified from *E. coli* cells was subjected to crystallization. A crystal was not made under the reported conditions for crystallization of authentic bovine F_1_ 27 probably because of the histidine‐tag of the β‐subunit of *E. coli*‐expressed bovine F_1_. After screening crystallization conditions, we found that crystals were reproducibly formed in the solution containing 0.5 mm AMPPNP and 20 mm MgCl_2_ with PEG3350 as precipitant (Fig. 3A). Crystals were collected from the drops, washed, and analyzed by SDS/PAGE (Fig. 3B). All the five F_1_ subunits were detected in the gel, confirming that the crystals were of bovine F_1_. The result shows that the purified bovine F_1_ has a quality high enough to grow crystals.

## Conclusions

Bovine F_1_, for the first time, was successfully expressed in *E. coli* cells. As expected, purified bovine F_1_ exhibits motor characteristics similar to those of human F_1_. It forms good crystals rather easily. We previously spent time and efforts for crystallization of bacterial F_1_ 28, but now realize that bovine F_1_ is superior to bacterial F_1_ in crystallization for the detailed structural study of F_1_. Availability of mutants adds a further advantage to *E. coli*‐expressed bovine F_1_ over the native protein.

Without AF1 and AF2, only very little, if any, bovine F_1_ was produced by *E. coli*, indicating that AF1 and AF2 are required for efficient production of bovine F_1_. This expression is the first demonstration of the chaperone function of these two factors for assembly of mammalian F_1_. As speculated in a previous yeast study 29, mammalian AF2 would bind to α‐subunit by mimicking the coiled‐coil region of the γ‐subunit and then, α‐ and β‐subunit eject their cognate chaperone factors by switching their partner on the way of the assembly. In relation to this, a metabolic disease with a decreased amount of F_o_F_1_ in mitochondria is attributed to a mutation in the *ATP12* gene, suggesting a critical physiological role of these assembly factors in production of functional F_o_F_1_ 30. Although we did not test human *ATP11* and *ATP 12* for expressing bovine F_1_, sequence similarities of AF1 and AF2 are 93% and 88% such that we would expect them to be interchangeable. We expect that the development and improvement of the present bovine F_1_ expression system would open a way to the study of detailed mechanisms of the assembly and a structure‐mechanism relationship of mitochondrial F_1_.

## Author contributions

TS and MY conceived and designed the experiments and wrote the paper. TS developed the *E. coli* expression system of bovine F_1_ with NI and JS and the X‐ray crystallographic system with YW and TE, and single‐molecule analytical system. TH gave critical suggestions for experimental systems and interpretations throughout this study.

## References

[1] Yoshida M , Muneyuki E and Hisabori T (2001) ATP synthase – a marvellous rotary engine of the cell. Nat Rev Mol Cell Biol 2, 669–677. 11533724 10.1038/35089509

[2] Boyer PD (2002) A research journey with ATP synthase. J Biol Chem 277, 39045–39061. 12181328 10.1074/jbc.X200001200

[3] Senior AE , Nadanaciva S and Weber J (2002) The molecular mechanism of ATP synthesis by F_1_F_0_‐ATP synthase. Biochim Biophys Acta 1553, 188–211. 11997128 10.1016/s0005-2728(02)00185-8

[4] Noji H , Yasuda R , Yoshida M and Kinosita K Jr (1997) Direct observation of the rotation of F_1_‐ATPase. Nature 386, 299–302. 9069291 10.1038/386299a0

[5] Suzuki T , Tanaka K , Wakabayashi C , Saita E and Yoshida M (2014) Chemomechanical coupling of human mitochondrial F_1_‐ATPase motor. Nat Chem Biol 10, 930–936. 25242551 10.1038/nchembio.1635

[6] Yasuda R , Noji H , Kinosita K Jr and Yoshida M (1998) F_1_‐ATPase is a highly efficient molecular motor that rotates with discrete 120 degree steps. Cell 93, 1117–1124. 9657145 10.1016/s0092-8674(00)81456-7

[7] Yasuda R , Noji H , Yoshida M , Kinosita K Jr and Itoh H (2001) Resolution of distinct rotational substeps by submillisecond kinetic analysis of F_1_‐ATPase. Nature 410, 898–904. 11309608 10.1038/35073513

[8] Adachi K , Oiwa K , Nishizaka T , Furuike S , Noji H , Itoh H , Yoshida M and Kinosita K Jr (2007) Coupling of rotation and catalysis in F_1_‐ATPase revealed by single‐molecule imaging and manipulation. Cell 130, 309–321. 17662945 10.1016/j.cell.2007.05.020

[9] Furuike S , Hossain MD , Maki Y , Adachi K , Suzuki T , Kohori A , Itoh H , Yoshida M and Kinosita K Jr (2008) Axle‐less F_1_‐ATPase rotates in the correct direction. Science 319, 955–958. 18276891 10.1126/science.1151343

[10] Walker JE and Dickson VK (2006) The peripheral stalk of the mitochondrial ATP synthase. Biochim Biophys Acta 1757, 286–296. 16697972 10.1016/j.bbabio.2006.01.001

[11] Bason JV , Montgomery MG , Leslie AG and Walker JE (2015) How release of phosphate from mammalian F_1_‐ATPase generates a rotary substep. Proc Natl Acad Sci USA 112, 6009–6014. 25918412 10.1073/pnas.1506465112 PMC4434703

[12] Ackerman SH (2002) Atp11p and Atp12p are chaperones for F_1_‐ATPase biogenesis in mitochondria. Biochim Biophys Acta 1555, 101–105. 12206899 10.1016/s0005-2728(02)00262-1

[13] Wang ZG and Ackerman SH (2000) The assembly factor Atp11p binds to the beta‐subunit of the mitochondrial F_1_‐ATPase. J Biol Chem 275, 5767–5772. 10681564 10.1074/jbc.275.8.5767

[14] Wang ZG , Sheluho D , Gatti DL and Ackerman SH (2000) The α‐subunit of the mitochondrial F_1_‐ATPase interacts directly with the assembly factor Atp12p. EMBO J 19, 1486–1493. 10747017 10.1093/emboj/19.7.1486 PMC310218

[15] Wang ZG , Schmid KJ and Ackerman SH (1999) The *Drosophila* gene 2A5 complements the defect in mitochondrial F_1_‐ATPase assembly in yeast lacking the molecular chaperone Atp11p. FEBS Lett 452, 305–308. 10386611 10.1016/s0014-5793(99)00676-6

[16] Wang ZG , White PS and Ackerman SH (2001) Atp11p and Atp12p are assembly factors for the F_1_‐ATPase in human mitochondria. J Biol Chem 276, 30773–30778. 11410595 10.1074/jbc.M104133200

[17] Hinton A , Zuiderweg ER and Ackerman SH (2003) A purified subfragment of yeast Atp11p retains full molecular chaperone activity. J Biol Chem 278, 34110–34113. 12829692 10.1074/jbc.M305353200

[18] Sheluho D and Ackerman SH (2001) An accessible hydrophobic surface is a key element of the molecular chaperone action of Atp11p. J Biol Chem 276, 39945–39949. 11522798 10.1074/jbc.M107252200

[19] Hinton A , Gatti DL and Ackerman SH (2004) The molecular chaperone, Atp12p, from Homo sapiens. *In vitro* studies with purified wild type and mutant (E240K) proteins. J Biol Chem 279, 9016–9022. 14701807 10.1074/jbc.M312631200

[20] Knowles AF and Penefsky HS (1972) The subunit structure of beef heart mitochondrial adenosine triphosphatase. Isolation procedures. J Biol Chem 247, 6617–6623. 4263201

[21] Suzuki T , Ueno H , Mitome N , Suzuki J and Yoshida M (2002) F_o_ of ATP synthase is a rotary proton channel. Obligatory coupling of proton translocation with rotation of *c*‐subunit ring. J Biol Chem 277, 13281–13285. 11815616 10.1074/jbc.M111210200

[22] Matsui T and Yoshida M (1995) Expression of the wild‐type and the Cys‐/Trp‐less α_3_β_3_γ complex of thermophilic F_1_‐ATPase in *Escherichia coli* . Biochim Biophys Acta 1231, 139–146. 7662694 10.1016/0005-2728(95)00070-y

[23] Jault JM , Dou C , Grodsky NB , Matsui T , Yoshida M and Allison WS (1996) The α_3_β_3_γ subcomplex of the F_1_‐ATPase from the thermophilic *Bacillus* PS3 with the betaT165S substitution does not entrap inhibitory MgADP in a catalytic site during turnover. J Biol Chem 271, 28818–28824. 8910526 10.1074/jbc.271.46.28818

[24] Walker JE , Fearnley IM , Gay NJ , Gibson BW , Northrop FD , Powell SJ , Runswick MJ , Saraste M and Tybulewicz VL (1985) Primary structure and subunit stoichiometry of F_1_‐ATPase from bovine mitochondria. J Mol Biol 184, 677–701. 2864455 10.1016/0022-2836(85)90313-4

[25] Bason JV , Runswick MJ , Fearnley IM and Walker JE (2011) Binding of the inhibitor protein IF1 to bovine F_1_‐ATPase. J Mol Biol 406, 443–453. 21192948 10.1016/j.jmb.2010.12.025 PMC3041923

[26] Suzuki T , Suzuki J , Mitome N , Ueno H and Yoshida M (2000) Second stalk of ATP synthase. Cross‐linking of γ subunit in F_1_ to truncated F_o_ *b* subunit prevents ATP hydrolysis. J Biol Chem 275, 37902–37906. 10970900 10.1074/jbc.M007075200

[27] Lutter R , Abrahams JP , van Raaij MJ , Todd RJ , Lundqvist T , Buchanan SK , Leslie AG and Walker JE (1993) Crystallization of F_1_‐ATPase from bovine heart mitochondria. J Mol Biol 229, 787–790. 8433373 10.1006/jmbi.1993.1081

[28] Shirakihara Y , Shiratori A , Tanikawa H , Nakasako M , Yoshida M and Suzuki T (2015) Structure of a thermophilic F_1_‐ATPase inhibited by an ε‐subunit: deeper insight into the ε‐inhibition mechanism. FEBS J 282, 2895–2913. 26032434 10.1111/febs.13329

[29] Ludlam A , Brunzelle J , Pribyl T , Xu X , Gatti DL and Ackerman SH (2009) Chaperones of F_1_‐ATPase. J Biol Chem 284, 17138–17146. 19383603 10.1074/jbc.M109.002568 PMC2719352

[30] De Meirleir L , Seneca S , Lissens W , De Clercq I , Eyskens F , Gerlo E , Smet J and Van Coster R (2004) Respiratory chain complex V deficiency due to a mutation in the assembly gene *ATP12* . J Med Genet 41, 120–124. 14757859 10.1136/jmg.2003.012047 PMC1735674

